# EZH2 expression correlates with locoregional recurrence after radiation in inflammatory breast cancer

**DOI:** 10.1186/s13046-014-0058-9

**Published:** 2014-07-23

**Authors:** Bisrat G Debeb, Yun Gong, Rachel L Atkinson, Nour Sneige, Lei Huo, Ana Maria Gonzalez-Angulo, Mien-Chie Hung, Vicente Valero, Naoto T Ueno, Wendy A Woodward

**Affiliations:** 1 Department of Radiation Oncology, The University of Texas M. D. Anderson Cancer Center, Houston, TX, USA; 2 Department of Pathology, The University of Texas M. D. Anderson Cancer Center, Houston, TX, USA; 3 Department of Clinical Cancer Prevention, The University of Texas M. D. Anderson Cancer Center, Houston, TX, USA; 4 Department of Breast Medical Oncology, The University of Texas M. D. Anderson Cancer Center, Houston, TX, USA; 5 Department of Molecular and Cellular Oncology, The University of Texas M. D. Anderson Cancer Center, Houston, TX, USA; 6 Morgan Welch Inflammatory Breast Cancer Research Program and Clinic, The University of Texas M.D. Anderson Cancer Center, Houston, TX, USA; 7 Division of Radiation Oncology, The University of Texas M.D. Anderson Cancer Center, 1515 Holcombe Blvd., Unit 1202, Houston, 77030, TX, USA; 8 Center for Molecular Medicine and Graduate Institute of Cancer Biology, China Medical University, Taichung, Taiwan

**Keywords:** Inflammatory breast cancer, EZH2, Radiation, Locoregional recurrence

## Abstract

**Background:**

Enhancer of zeste homolog 2 (EZH2), a member of the polycomb group proteins, has been shown to promote cancer progression and breast cancer stem cell (CSC) expansion. Breast CSCs are associated with resistance to radiation in inflammatory breast cancer (IBC), a rare but aggressive variant of breast cancer. In this retrospective study, we examined the clinical role of EZH2 in locoregional recurrence (LRR) of IBC patients treated with radiation.

**Patients and methods:**

62 IBC patients who received radiation (7 pre-operative, 55 post-operative) and had adequate follow up to assess LRR were the subject of this study. Positive EZH2 status was defined as nuclear immunohistochemical staining in at least 10% of invasive cancer cells. Association of EZH2 expression with clinicopathologic features were evaluated using the Chi-square statistic and actuarial LRR free survival (LRFS) was determined using the Kaplan-Meier method.

**Results:**

The median follow-up for this cohort was 33.7 months, and the 5-year overall LRFS rate was 69%. Of the 62 patients, 16 (25.8%) had LRR, and 15 out of 16 LRR occurred in EZH2 expressing cases. Univariate analysis indicated that patients who had EZH2-positive IBC had a significantly lower 5-year locoregional free survival (LRFS) rate than patients who had EZH2-negative IBC (93.3% vs. 59.1%; P = 0.01). Positive EZH2 expression was associated significantly with negative ER status (97.1% in ER- vs 48.1% in ER+; P < 0.0001) and triple-negative receptor status (P = 0.0001) and all triple-negative tumors were EZH2-positive. In multivariate analysis, only triple negative status remained an independent predictor of worse LRFS (hazard ratio 5.64, 95% CI 2.19 – 14.49, P < 0.0001).

**Conclusions:**

EZH2 correlates with locoregional recurrence in IBC patients who received radiation treatment. EZH2 expression status may be used in addition to receptor status to identify a subset of patients with IBC who recur locally in spite of radiation and may benefit from enrollment in clinical trials testing radiosensitizers.

## Introduction

The use of ionizing radiation is an integral component of breast cancer treatment for all patients who receive breast conserving surgery and in most patients with locally advanced breast cancer. Resistance to radiation is, however, a common reason for local recurrence in breast cancer patients, especially in breast cancers with high risk of recurrence such as inflammatory and triple-negative breast cancers [1],[2]. Recurrence is thought to be driven in part by tumor initiating cells or cancer stem cells (CSCs), a subpopulation of self-renewing cancer cells which exhibit tumor initiating properties and have been shown to contribute to the development of resistance to radiation and chemotherapy. Our lab and others have provided evidence that breast CSCs are resistant to radiation [3]–[5] although detailed mechanisms of resistance have yet to be fully investigated.

Inflammatory breast cancer (IBC) is a rare but aggressive variant of invasive breast cancer characterized by rapid progression, enlargement of the breast, skin edema and erythema. Typically, IBC is associated with rapid metastasis, resistance to treatment, and poor prognosis–all hallmarks of the CSC hypothesis. To date clinical and preclinical data strongly correlate CSCs with IBC [6]. Despite advances in multimodal breast cancer care, the clinical outcome of these patients remains poor demonstrating a critical need to identify novel therapeutics that target the distinct biology of IBC. A recent study by Gong and colleagues [7] showed that Enhancer of zeste homolog 2 (EZH2), a member of the polycomb group proteins, is expressed very frequently in IBC and is associated with worse clinical outcome in these patients. This work was supported by in vitro findings that EZH2 is expressed at higher levels in human IBC cell lines and its knockdown suppresses growth and invasion in IBC cells [8]. Previous studies have shown that EZH2 is involved in maintaining the self-renewal capability of adult and embryonic stem cells [9],[10] and recently, Chang et al has demonstrated that EZH2 promotes the expansion of breast CSCs and that it impairs DNA repair in breast cancer cells by specific downregulation of RAD51 gene [11]. In addition, they showed that HIF1α, a known mediator of radiation resistance, transactivated the EZH2 gene and increased EZH2 expression under hypoxic conditions [11]. These findings suggest a possible involvement of EZH2 in radioresistance, however, the clinical role of EZH2 in local failure and radiation resistance in breast cancer patients is unknown. Herein, we investigated the relation between EZH2 expression and locoregional failure and found that positive EZH2 expression correlates with lower locoregional recurrence free survival after radiation in IBC patients.

## Materials and methods

**Figure 1:**
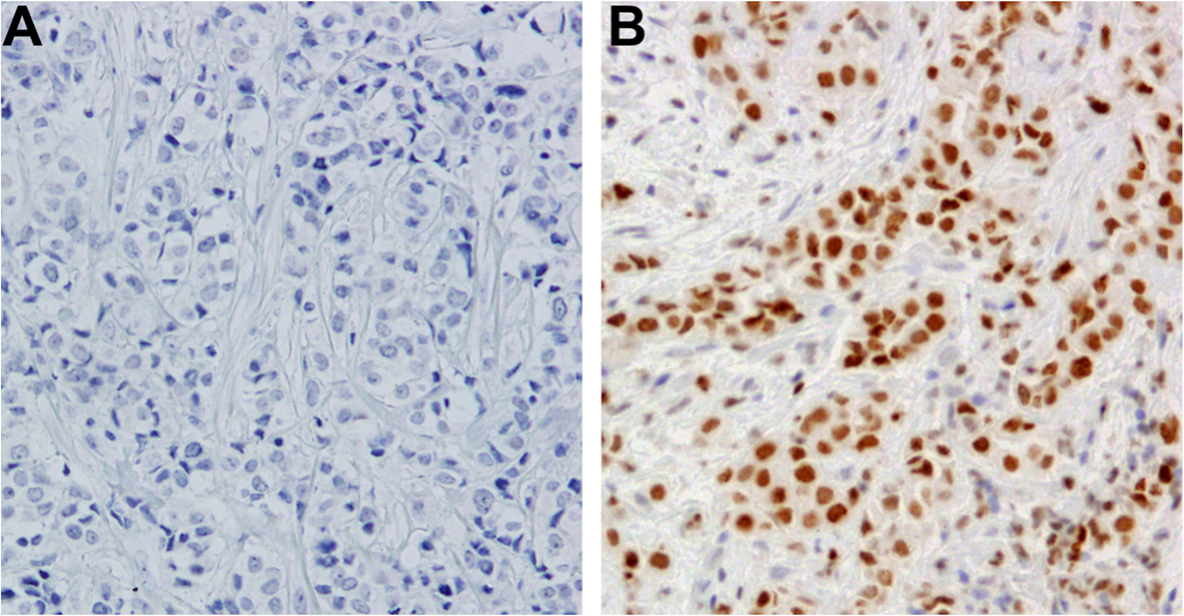
Representative images for immunohistochemical staining of EZH2 in IBC tumors (A) EZH2-negative IBC tumor (B) EZH2-positive IBC tumor.

This study was approved by The University of Texas MD Anderson Cancer Center Institutional Review Board. The diagnosis, preoperative and postoperative treatments of these patients, biomarker study (encompassing ER, PR, and HER2 status), and tissue microarray (TMA) construction using post-neoadjuvant residual tumors as well as EZH2 immunohistochemical staining and evaluation were previously reported [7]. EZH2 staining was interpreted and recorded independently by 2 pathologists (Y.G. and L.H.) in a blinded manner. Positive EZH2 status was defined as nuclear staining in at least 10% of invasive cancer cells. Images of negative and positive EZH2 staining results in representative tumors are shown in Figure 1. To evaluate the role of EZH2 in radiation resistance, the radiation record of all patients was re-reviewed and only patients who received radiation (62 patients) were included in this study. Patients who had local failure prior to receiving radiation were excluded from this analysis.

### Statistical analysis

Chi-square or Fisher exact test was used to evaluate associations between EZH2 status and clinicopathologic variables. We used the Kaplan-Meier method to estimate actuarial LRR free survival (LRFS). LRFS was calculated from the date of initial pathologic diagnosis of the primary tumor to the date of locoregional recurrence or the date of last follow-up and any locoregional recurrence was considered an event. A Cox proportional hazards regression model was then used to test the statistical significance of several potential prognostic factors for LRFS. The factors analyzed included EZH2 expression; age; race; lymph node status; histologic type; lymphovascular invasion; ER, PR, and HER-2 status; triple-negative (ER-negative, PR-negative, and HER2 negative) status; and twice-a-day (BID) radiation. This modeling was done in a univariate fashion. Then, all potential prognostic factors with a P value < .25 from the univariate analysis were included in a saturated model, and backward elimination was used to remove factors from the model based on the likelihood ratio test in the multiple regression analysis. All statistical analysis was conducted using StataCorp. 2011. Stata Statistical Software: Release 12. College Station, TX: StataCorp LP. All reported P values were 2-sided, and P < 0.05 was considered statistically significant.

## Results

The cohort of IBC patients used in this study and the EZH2 expression were described previously [7]. Briefly, tumors from 88 patients with primary IBC were included of which EZH2 staining was available for 74 tumors. Patients received multimodal treatment, including neoadjuvant chemotherapy, surgery, and radiation therapy. At the completion of neoadjuvant chemotherapy, all patients underwent mastectomy; most patients also underwent axillary lymph node dissection. Thirty-one patients received adjuvant endocrine therapy, and all patients with HER2-positive tumors received adjuvant trastuzumab. Timing of radiation was explicitly reviewed for this study. Seven patients previously classified as post-op radiation [7] were noted to have had pre-operative radiation. In total, sixty-two patients received radiation (7 pre-operative, 55 post-operative) to the chest wall and draining lymphatics. In this study, we reviewed the radiation record of these patients to evaluate the role that EZH2 plays in mediating radiation resistance in clinically radioresistant IBC.

In the 62 patients who received radiation, the median follow-up was 33.7 months (range, 1.1-181.5 months). The age at the time of initial diagnosis ranged from 23 years to 75 years (median age, 48 years). Forty-eight (81.3%) were whites, 8 (13.5%) were Hispanics, and 3 (5.1%) were Blacks and Asians. Lymph node involvement was observed in 53 of 60 patients (88.3%). Histologically, 54 of 62 tumors (87.1%) were ductal type, and 47 of 56 (83.9%) demonstrated lymphovascular invasion. Positive ER expression was observed in 27 of 61 of tumors (44.2%), positive PR status was observed in 19 of 61 tumors (31.1%), and HER2 overexpression and/or amplification was observed in 22 of 61 tumors (36.1%). Triple-negative status was observed in 16 of 61 tumors (26.2%). Thirty-eight of 48 (79.2%) IBC patients received BID radiation, of which 37 (77.1%) received a total dose of 63.47 Gy and the remaining received a dose of 67.09 Gy.

### EZH2 expression and clinicopathologic variables in patients who received radiation

In this cohort positive EZH2 expression was associated significantly with negative ER status (P < 0.0001) and triple-negative status (P = 0.005). Specifically EZH2 was expressed more frequently in ER-negative (97.1%; 33 of 34 ER- tumors) than ER-positive patients (48.1%; 13 of 27 ER + tumors) treated with radiation (Table 1). All triple-negative tumors were EZH2-positive. EZH2 expression was not associated significantly with lymphnode status, histologic type, lymphovascular invasion, PR status, HER2 status and BID radiation (Table 1). Similar results were found when only post mastectomy radiation-treated IBC patients were analyzed (data not shown).

**Table 1:** Comparison of clinicopathological parameters in women who received radiation stratified by EZH2 expression

**Prognostic factors**	**EZH2 negative**		**EZH2 positive**		
	**Number of patients**	**Percent**	**Number of patients**	**Percent**	**P value**
Age of diagnosis (N = 62)					
≥ 45	11	68.75	29	63.04	0.77
< 45	5	31.25	17	39.96	
Race (N = 59)					
Non-Hispanic White	15	93.75	33	76.74	0.26
All others	1	6.25	10	23.26	
Lymph node status (N = 60)					
Negative	3	18.75	4	9.09	0.23
Positive	13	81.25	40	90.91	
Histologic type (N = 62)					
Ductal	12	75.0	42	91.30	0.19
Others	4	25.0	4	8.70	
Lymphovascular invasion (N = 56)					
No	4	26.67	5	12.20	0.23
Yes	11	73.33	36	87.80	
ER expression (N = 61)					
Negative	1	6.67	33	71.74	< 0.0001
Positive	14	93.33	13	28.26	
PR expression (N = 61)					
Negative	8	53.33	34	73.91	0.19
Positive	7	46.67	12	26.09	
HER2 expression (N = 61)					
Negative	11	73.33	28	60.87	0.54
Positive	4	26.67	18	39.13	
Triple-negative status (N = 61)					
No	15	100.00	30	65.22	0.005
Yes	0	0.00	16	34.78	
Radiation type (N = 62)					
Preoperative	1	6.25	6	13.04	0.66
Postoperative	15	93.75	40	86.96	
BID radiation (N = 48)					
No	0	0.00	10	26.32	0.09
Yes	10	100.00	28	73.68	
Radiation dose (N = 48)		Dose		Dose	
	11	67.09	37	63.47	0.03

### EZH2 expression and local failure

**Figure 2:**
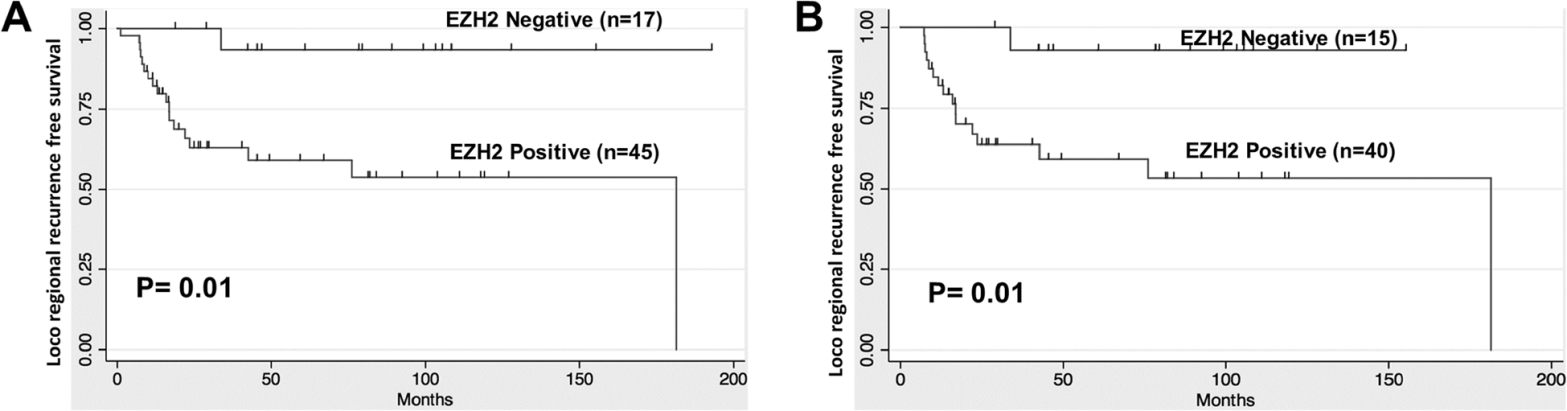
**Kaplan Meier curve showing that EZH2 is associated with lower LRFS in IBC patients. A)** All patients who received pre- and post-operative radiation treatment (N = 62) and **B)** Postmastectomy radiation cohort (N = 55) showed that the LRFS in EZH2 negative cases was significantly higher than in EZH2-positive cases (P = 0.01).

Of the 62 patients who had follow-up information available on LRR, the median LRFS duration was 4.04 years (95% CI, 2.85-8.79 years). The 5-year LRFS rate for the entire cohort of patients was 69% (Figure 2). Sixteen (25.8%) had LRR and notably 15 of the 16 LRR occurred in EZH2 positive patients. In univariate analysis, positive EZH2 expression was associated significantly with a lower LRFS rate (P = 0.01) (Figure 2). The 5-year LRFS rate for patients who had EZH2-positive tumors was 59.1% compared with 93.3% for patients who had EZH2-negative tumors (Figure 2A). Among the 55 patients who had post mastectomy radiation, positive EZH2 expression was also significantly associated with lower LRFS rates (5-year LRFS EZH2-positive = 59.4%, EZH2-negative = 92.9%, P = 0.01; Figure 2B).

Univariate analyses were performed to determine whether any other clinicopathologic factors were associated with the clinical outcome of IBC patients. We observed that lower LRFS rates were associated significantly with negative ER status (P = 0.001) and with triple-negative status (Table 2; P = 0.0001). There was no significant association between LRFS rates and histologic type, age, race, lymph node status, and HER2 status while there was a trend with lymphovascular invasion (P = 0.07). In multivariate analysis, we observed that only triple negative status remained an independent predictor of LRFS (hazard ratio 5.64, 95% CI 2.19 – 14.49, P < 0.0001; Table 3). We reasoned that failure of EZH2 to predict local recurrence was influenced by triple negative-receptor-status since all of the triple-negative tumors were EZH2-positive. Thus, we excluded triple negative tumors from the analysis and we found that EZH2 has a trend to be an independent predictor of worst LRFS in the 45 IBC patients analyzed (6.57, 95% CI 0.82-52.87; P = 0.08) (Table 4).

**Table 2:** Relation between LRFS, EZH2 and clinicopathologic factors in patients who received radiation

**Prognostic factors**	**Number of patients/number of deaths**	**5-year LRFS (95% CI)**	**P value**
Age of diagnosis (N = 62)			
≥ 45	40/12	72.7 (54.8 – 84.8)	0.43
< 45	22/7	60.9 (33.9 – 79.6)	
Race (N = 59)			
Non-Hispanic White	48/13	74.3 (58.4 – 85.1)	0.36
All others	11/4	56.1 (19.5 – 81.5)	
Lymph node status (N = 60)			
Negative	7/2	83.3 (27.3 – 97.4)	0.79
Positive	53/16	67.3 (51.3 – 79.2)	
Histologic type (N = 62)			
Ductal	54/17	68.7 (53.2 – 80.1)	0.72
Others	8/2	75.0 (31.5 – 93.1)	
Lymphovascular invasion (N = 56)			
No	9/0	100	0.07
Yes	47/16	66.8 (48.9 – 78.5)	
ER expression (N = 61)			
Negative	34/16	44.4 (24.1 – 62.9)	0.001
Positive	27/3	92.3 (72.6 – 98.0)	
PR expression (N = 61)			
Negative	42/16	58.4 (39.9 – 73.0)	0.025
Positive	19/3	88.2 (60.2 – 96.9)	
HER2 expression (N = 61)			
Negative	39/13	68.5 (49.9 – 81.2)	0.81
Positive	22/6	70.0 (39.1 – 84.3)	
Triple-negative status (N = 61)			
No	45/9	82.6 (66.6 – 91.4)	0.0001
Yes	16/10	25.7 ( 6.4 – 51.0)	
Radiation type (N = 62)			
Postoperative	55/17	69.4 (54.0 – 80.5)	0.73
Preoperative	7/2	64.3 (15.2 – 90.2)	
BID radiation (N = 48)			
No	10/3	80.0 (40.9 – 94.6)	0.21
Yes	38/14	58.0 ( 38.9 – 73.0)	
EZH2 (N = 62)			
No	17/1	92.8 (59.1 – 98.9)	0.01
Yes	45/18	59.2 (41.5 – 73.1)	

**Table 3:** Multivariate Cox model for LRFS in patients who received radiation

	**Hazard ratio (95% CI)**	**P value**
Triple negative status	5.64 (2.19 – 14.49)	<0.0001

**Table 4:** Multivariate Cox model for LRFS in patients who received radiation but excluding those with triple negative receptor status

	**Hazard ratio (95% CI)**	**P value**
EZH2	6.5 (0.82 – 52.75)	0.077

## Discussion

Herein, we report that EZH2 expression correlates with locoregional recurrence in IBC patients who received radiation. Although EZH2 is associated with local failure after radiation in univariate analyses, it is not independently associated with local failure, in part because nearly all patients with ER-negative disease overexpress EZH2, making it impossible to separate the influences of EZH2 expression and receptor negativity. When examining the influence in non-triple negative cohort, however, EZH2 expression trends to be an independent predictor of locoregional recurrence. As such EZH2 ER + patients may be appropriately included in studies of radiosensitizers for high risk IBC.

The clinical-pathological features of IBC include enrichment of factors that have been previously associated with radioresistant disease, including negative receptor status and a phenotype enriched for radioresistant breast CSCs [6],[12],[13]. IBC is known to be associated with a high incidence of locoregional recurrence [12],[14], and thus, identification of markers and a greater understanding of the biology of radiation resistance in IBC will be desirable in order to develop novel radiosensitizers that could improve local control in IBC patients. The clinical role of EZH2 in radiation resistance has not been reported before. However, several studies have suggested the possible involvement of EZH2 in radiation resistance. Recent evidence from Hung’s group suggests that enhanced expression of EZH2 promotes breast CSC expansion through impairment of the DNA damage repair protein Rad51 and the activation of RAF1-ERK-β-catenin signaling [11]. They showed that EZH2-mediated downregulation of DNA damage repair leads to accumulation of recurrent *RAF1* gene amplification in breast CSCs, which activates p-ERK-β-catenin signaling to promote CSC expansion. They further revealed that targeting EZH2 downstream activation pathways such as RAF1-ERK signaling with the MEK inhibitor AZD6244 could prevent breast cancer progression by eliminating CSCs. They further showed that HIF1α, a known mediator of radioresistance in breast cancer, activates the *EZH2* gene and increases EZH2 expression under hypoxic conditions [11]. Other studies have also supported the possible role for EZH2 in modulating radiation response. Dong et al demonstrated that overexpression of Bmi-1, another PcG protein similar to EZH2, elicits radioprotective effects in keratinocytes by mitigating the genotoxic effects of radiation through epigenetic mechanisms [15]. In another study, pharmacologic inhibition of EZH2 induced radiation sensitivity in atypical teratoid/rhabdoid tumors in vitro [16], and silencing EZH2 with RNAi enhanced radiation sensitivity in lung cancer cells [17]. Collectively, these data together with our current findings that EZH2 is associated with local failure in IBC patients support the hypothesis that EZH2 has a significant role in promoting resistance to radiation treatment. However, it remains unknown which, if any, of the known mechanisms of EZH2 activity actually modulates resistance to radiation therapy.

We and others have provided evidence that breast CSCs are resistant to radiation through upregulation of stem cell self renewal pathways including β-catenin and Notch signaling [3],[4] and other studies have shown that CSCs contribute to radioresistance by preferential activation of the DNA damage checkpoint response and increased DNA repair capacity and by maintaining low ROS levels [18],[19]. EZH2 has been shown to promote CSC expansion and maintenance [11],[20] and to impair DNA repair via downregulation of Rad51 [11],[21]. These findings seem paradoxical given that downregulation of Rad51 is expected to increase radiosensitivity but CSC expansion has been linked with radiation resistance. Further studies are warranted to elucidate this paradox by examining how EZH2 activates radiation resistance mechanisms in breast cancer cells.

It is to be noted that the tumors included in this study comprised tissues from refractory or residual tumors after neoadjuvant systemic therapy. Previous studies have shown that neoadjuvant chemotherapy increased the CSC subpopulation [22] and that EZH2 promotes the expansion of CSCs [11],[20]. It is possible then that the expression of EZH2 described in this cohort is influenced by neoadjuvant chemotherapy. This should be considered in future studies.

## Conclusion

In conclusion, this retrospective study showed that EZH2 is associated with receptor-negative status and lower locoregional-recurrence free survival rates in IBC patients. Additional examination of the mechanism of this clinical finding and its association with triple negative receptor status is warranted. These findings indicate that EZH2 expression status may be used in conjunction with ER + status to identify a subset of patients with IBC who recur locally in spite of radiation and may benefit from enrollment in clinical trials testing radiosensitizers. Given the high frequency of expression of EZH2 and local recurrence in IBC patients, targeting EZH2 may provide a novel therapeutic strategy to improve local failure of patients with IBC.

## Competing interests

The authors have no competing interests to disclose.

## Authors’ contributions

Collection and/or assembly of data: BGD, YG, RLA, WAW; provided and/or characterized patient tissue samples: YG, LH, NS, AMG, MH, VV, NTU, WAW; data analysis and interpretation: BGD, YG, RLA, LH, WAW; Manuscript writing: BGD, YG, and WAW; Final approval of manuscript by all authors.
